# Development and Evaluation of an iiPCR Assay for *Salmonella* and *Shigella* Detection on a Field-Deployable PCR System

**DOI:** 10.1155/2020/9373984

**Published:** 2020-09-07

**Authors:** Tian Du, Ji-hong Lin, Jun-hua Zhao, Hai-bo Wang, Qiu-hua Mo

**Affiliations:** ^1^Futian District Center for Disease Control and Prevention, Shenzhen 518040, Guangdong, China; ^2^Zhongshan International Travel Healthcare Center, Zhongshan 528400, Guangdong, China; ^3^State Key Laboratory of Diarrhea Disease Detection, Zhuhai International Travel Healthcare Center, Zhuhai 519020, Guangdong, China

## Abstract

**Background:**

*Salmonella* and *Shigella* are often associated with fecal-oral transmission and cause large-scale outbreaks in centralized catering units and, therefore, should be frequently and strictly monitored, especially among food handlers. However, no specific and sensitive on-site detection method is available until now.

**Methods:**

In this study, an insulated isothermal PCR assay for the detection of *Salmonella* and *Shigella* on a field-deployable PCR system was developed. Specificity, sensitivity, reproducibility, and clinical accuracy of the assay were characterized and evaluated.

**Results:**

The insulated isothermal PCR assay could be completed within 58 minutes with minimal pretreatment needed. The assay was specific and with good reproducibility. The limit of detection was 10^3^ CFU/mL and 10^1^ CFU/mL for *Salmonella* and *Shigella*, respectively, which was comparable to multiplex real-time PCR. Mock on-site clinical evaluation results showed that the analytical sensitivity and specificity of the insulated isothermal PCR assay were 100% and 96.6%, while the positive predictive value and negative predictive value were 94.1% and 100%, respectively.

**Conclusion:**

Based on our results, we believe that the assay developed herein could serve as an alternative method for preliminary screening and provide a valuable platform for the on-site detection of *Salmonella* and *Shigella*, especially in resource-limited and developing countries.

## 1. Introduction

Diarrhea is an ordinary disease but with a relatively high morbidity and mortality in developing countries according to recent reports [1, 2]. Once infected, patients may suffer from the symptoms for days and spread the disease to others, which caused high socioeconomic burden [3]. Generally, the causative agent for infectious diarrhea may be viruses (such as norovirus and rotavirus), parasites (such as *Giardia lamblia* and *Entamoeba histolytica*), or bacteria (such as *Salmonella* and *Shigella*). Among these causative agents, *Salmonella* and *Shigella* are often associated with fecal-oral transmission and cause large-scale outbreaks in centralized catering units [4, 5]. Therefore, as a preventive action, governments issued laws for obligatory *Salmonella* and *Shigella* screening among food handlers.

The gold standard for the screening and detection of *Salmonella* and *Shigella* is culture. However, many hands-on steps are needed which make the method labor-intensive and time-consuming. Moreover, the lower limit of detection (LOD) of the method may be as high as 10^4^ CFU/mL [6]. With the aim of improving the effectiveness and sensitivity, we developed several nucleic acid-based assays, including PCR [6, 7], DNA microarray [8], and real-time nucleic acid sequence-based amplification (RT-NASBA) [9], for the detection of *Salmonella* and *Shigella* or other diarrhea-related pathogens. However, these assays are not suitable for on-site detection, where no sophisticated thermal cycler is available. Zhuang et al. developed the loop-mediated isothermal amplification (LAMP) assay for the on-site detection of *Salmonella* [10], while false-positive results may be generated due to the fact that the detection of LAMP products was via either turbidity or generic fluorescent dyes.

Recently, a fluorescent probe hydrolysis-based insulated isothermal PCR (iiPCR) technology was invented and commercially available [11]. With the field-deployable PCR machine (POCKIT Nucleic Acid Analyzer, GeneRadar Biotechnology Corp., Xiamen, China), on-site detection becomes possible. In our previous study, we have established a reverse transcription-iiPCR method for rapid on-site detection of yellow fever virus. The detection can be completed within one hour automatically with analytical specificity and sensitivity up to 100% and 92.31%, respectively [12]. In the current study, an iiPCR assay for on-site detection of *Salmonella* and *Shigella* on this field-deployable PCR system was developed and its specificity, sensitivity, and reproducibility were evaluated. Stool samples were used to evaluate the accuracy of the assay for its potential clinical application.

## 2. Materials and Methods

### 2.1. Samples

Human stool samples were collected using a stool tube from food handlers who came to our center for obligatory *Salmonella* and *Shigella* screening during 2017–2019 and first diagnosed by the bacterial culture method as described previously [6]. 183 samples, including 36 samples positive for *Salmonella*, 28 samples positive for *Shigella,* and 119 negative samples, were selected randomly. These samples were then used to evaluate the iiPCR assay. The study was approved by the ethics committee of the Zhuhai International Travel Healthcare Center and in compliance with the Helsinki Declaration. Informed consent was obtained from each study participant.

### 2.2. Primers and Probes

Primers and probes used in the current study are listed in Table 1. Before designing the primers and probes, relevant sequences of *Salmonella* and *Shigella* in the GenBank were aligned and the conserved *ssaR* gene and *ipaH* gene were selected, respectively. The primers and probe sequences were generated using PrimerSelect software and further aligned with other pathogens, including *Vibrio*, *Staphylococcus*, and *Campylobacter*, to avoid the possibility of any undesired match. The probes were designed according to the principles recommended for the iiPCR (http://www.iipcr.com/index.php?func=faq), including moderate GC content and shorter length. The amplicons were analyzed with the MFold program (http://unafold.rna.albany.edu/?q=mfold) to avoid those amplicons with major secondary structures.

### 2.3. The iiPCR Assay

After several rounds of optimization through adjusting the concentration of buffer, primers, and probes, the iiPCR was established as follows. The reaction mixture, in R-Tube (GeneRadar Biotechnology Corp., Xiamen, China), contained 25.0 *μ*L Uni-ii buffer A, 1.5 *μ*L iiPCR Taq DNA Polymerase (5 U/*μ*L), 0.25 *μ*L tetramethylene sulfoxide, 0.5 *μ*L trehalose, 0.6 *μ*M of each primers (Sal-F, Sal-R, Shi-F, Shi-R), 0.3 *μ*M of each probes (Sal-P, Shi-P), and 5 *μ*L DNA template. After a short centrifugation on cubee™ microcentrifuge (GeneRadar Biotechnology Corp., Xiamen, China), the R-Tube was loaded onto the POCKIT device. A default program of 50°C for 10 min and 95°C for 48 min was conducted. Fluorescent signals were collected and signal-to-noise (S/N) ratios were calculated through dividing signals collected after iiPCR by those from before iiPCR [11]. Results were displayed on the screen automatically as “+” (Positive), “−” (Negative), or “?” (Unknown), using the default S/N thresholds.

### 2.4. Characterization of the Assay

Before specificity, sensitivity, and reproducibility evaluation, *Salmonella* (AB91111) and *Shigella* (AB200061) were cultured overnight in nutrient broth (LandBridge, Beijing, China) at 36°C and harvested for concentration determination by plate counting technique on XLD as described previously [13]. The specificity of the iiPCR assay was evaluated by detection of *Salmonella, Shigella,* and other related pathogens, including *Vibrio parahaemolyticus*, *Staphylococcus aureus*, norovirus GII, rotavirus A, *Giardia lamblia*, *Campylobacter jejuni, Yersinia enterocolitica,* and *Vibrio cholera* O1. The sensitivity of the iiPCR assay was evaluated by serial diluted *Salmonella* and *Shigella* and expressed as the limit of detection (LOD). The reproducibility of the iiPCR assay was evaluated through detection of 10^6^ CFU/mL (high concentration) and 10^4^ CFU/mL (low concentration) of both *Salmonella* and *Shigella* standard strains five times by two different operators.

### 2.5. Mock On-Site Evaluation of the Assay

183 samples (including both positive and negative samples as diagnosed by bacterial culture) were randomly selected and labeled blindly. To mimic on-site detection, a soybean-sized stool sample was first resuspended in 0.5 mL buffered saline, stood at room temperature for 5 min, and the supernatant was then boiled at 100°C for 5 min, which was used as the template for iiPCR. To minimize the heterogeneity problem of the samples, adjacent soybean-sized stool samples were collected for culture simultaneously. The analytical sensitivity and specificity, as well as the positive predictive value (PPV) and negative predictive value (NPV), were calculated as described previously [14].

## 3. Results

### 3.1. Characterization of the iiPCR Assay

**Figure 1:**
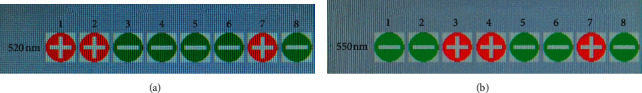
Representative results displayed during specificity evaluation. (a) and (b) Specificity analysis for *Salmonella* and *Shigella,* respectively. Samples 1 to 8 were *Salmonella* (AB91111, AB91018), *Shigella* (AB200061, AB200060), *Vibrio parahaemolyticus* (ATCC17802), *Staphylococcus aureus* (ATCC25923), positive control, and negative control.

**Figure 2:**
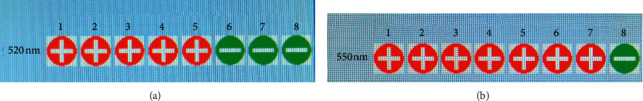
Representative results displayed during sensitivity evaluation. (a) and (b) Sensitivity analysis for *Salmonella* and *Shigella,* respectively. Samples 1 to 8 were *Salmonella* and *Shigella* from 10^7^ CFU/mL to 10^0^ CFU/mL.

After establishment, we evaluated the specificity and sensitivity of the iiPCR assay. Both evaluations performed in triple and 100% detection endpoints were shown. As shown in Figures 1(a) and 1(b), only *Salmonella* and *Shigella* could be detected at the 520 nm and 550 nm channel, respectively, while *Vibrio parahaemolyticus* and *Staphylococcus aureus* could not be detected. Other pathogens, including norovirus GII, rotavirus A, *Giardia lamblia*, *Campylobacter jejuni, Yersinia enterocolitica,* and *Vibrio cholera* O1, were also negative (data not shown). These results indicated the high specificity of the iiPCR assay. The sensitivity (as shown by LOD) of the iiPCR assay was determined by detecting serially diluted *Salmonella* and *Shigella* standard strains. As shown in Figures 2(a) and 2(b), the LOD for *Salmonella* and *Shigella* was 10^3^ CFU/mL and 10^1^ CFU/mL, respectively. The reproducibility of the iiPCR assay was evaluated through repeated detection of both high and low concentration of *Salmonella* and *Shigella.* Results showed that *Salmonella* and *Shigella* could be detected in all ten tests, suggesting good reproducibility of the assay (data not shown).

### 3.2. Mock On-Site Evaluation of the iiPCR Assay

In mock on-site evaluation using 183 stool samples, a total of 36 samples positive for *Salmonella* and 28 samples positive for *Shigella* were detected by both iiPCR assay and culture. However, four samples which were positive by iiPCR assay were tested to be negative by culture. Therefore, using culture as the gold standard, the calculated analytical sensitivity and specificity of the iiPCR assay were 100% and 96.6%, while the PPV and NPV were 94.1% and 100%, respectively (Table 2).

## 4. Discussion

Since *Salmonella* and *Shigella* are fecal-oral transmitted and cause large-scale outbreaks in centralized catering units, it is of great importance to frequently and strictly monitor food handlers. However, no specific and sensitive on-site detection method is available until now. In the current study, an iiPCR assay for the detection of *Salmonella* and *Shigella* on a field-deployable PCR system was developed and evaluated.

The specificity, sensitivity, and reproducibility characterization of the iiPCR assay were performed. To obtain high specificity, the primers/probes were designed within the most conserved regions of the genome of *Salmonella* and *Shigella* and further aligned with other related pathogens. Therefore, the iiPCR assay showed great selective ability for *Salmonella* and *Shigella* without cross-reaction with *Vibrio parahaemolyticus*, *Staphylococcus aureus*, norovirus GII, rotavirus A, *Giardia lamblia*, *Campylobacter jejuni, Yersinia enterocolitica,* and *Vibrio cholera* O1 (Figure 1). The sensitivity (as shown by LOD) for *Salmonella* and *Shigella* was 10^3^ CFU/mL and 10^1^ CFU/mL, respectively. This result is comparable to both our and other previous reports. In our previous study, the LOD for *Salmonella* and *Shigella* was 10^3^ CFU/mL and 10^1^ CFU/mL, respectively [6]. In other duplex assays, Cunningham et al. reported LOD of 990 CFU/mL and 52 CFU/mL, while Van Lint et al. reported LOD of 5528 CFU/mL and 61 CFU/mL for *Salmonella* and *Shigella*, respectively [15, 16]. During reproducibility characterization, *Salmonella* and *Shigella* could be detected in repeated tests by different operators, suggesting good reproducibility of the assay, which was an important parameter for clinical usage. When compared to the culture method using a national standard (which was also similar to standard ISO procedures), there was no difference with regard to specificity and reproducibility. However, the LOD of the culture method was 10^4^ CFU/mL and 10^3^ CFU/mL for *Salmonella* and *Shigella*, respectively [6], which was much higher than the iiPCR assay.

The mock on-site evaluation of the iiPCR assay was conducted using stool samples. The assay showed extremely high analytical sensitivity, specificity, PPV, and NPV, which indicated that the assay could be used as an efficient tool for the detection of *Salmonella* and *Shigella*. The four samples which were iiPCR positive and culture negative may contain viable but nonculturable (VBNC) *Salmonella*, as indicated by previous reports [17], or contain dead microorganisms. Temperature shift methods, including 56°C for 15 sec, 37°C for 3 days, or 30°C for 2 days, as indicated by previous studies [18–20], had been applied; however, they were still could not be cultured. As resuscitation-promoting stimuli (pyruvate and *α*-ketobutyrate), as suggested by Morishige et al. [21], were not available currently, whether they were in real VBNC state or just dead microorganisms needed further investigation. If some cases with clear clinical symptoms showed negative culture results, other serological tests should be conducted to confirm the results.

There are several advantages of this iiPCR assay. Unlike the PCR assay which needs a complicated and time-consuming nucleic acid extraction step, the iiPCR assay needs minimal pretreatment for samples (resuspend and boil), which is eligible for on-site detection. Moreover, lyophilized reagents, which were formatted for easy shipping at ambient temperature and storage, were used during the assay, and the field-deployable PCR machine (31-26-15 cm (W-D-H); 2.1 kg) can be packed into a suitcase and operated using a rechargeable battery (detailed information could be obtained from http://www.genereach.com). Since everything is packed, the detection could be taken place anytime and anywhere, which is highly suitable in resource-limited areas where conventional PCR facilities are lacking and in developing countries where stable power supply is not available. Furthermore, since the iiPCR was used to detect nucleic acid, theoretically it could effectively detect the nucleic acid from any kinds of samples, including food or environmental samples [22]. All these advantages made the iiPCR assay an easy-to-use tool for the detection of *Salmonella* and *Shigella*. There were also few disadvantages of this iiPCR. Firstly, now the iiPCR system is a qualitative system without quantitative module, which could not give the absolute quantification of the pathogens detected. However, its optical system is similar to the real-time PCR machine as stated by the producer. Therefore, it has the potential to be upgraded to a quantitative system in the near future. Secondly, the iiPCR, similar to other nucleic acid detection methods, could only detect targeted bacteria (*Salmonella* and *Shigella* in the current assay), while other bacteria which could also cause diarrhea are ignored [23]. Thirdly, the assay could not distinguish live or dead microorganisms as indicated in the current study. However, as an alternative method for preliminary screening, the iiPCR is acceptable. If the result is positive, other tests could be further conducted for confirmation.

## 5. Conclusion

In conclusion, an iiPCR assay for the detection of *Salmonella* and *Shigella* on a field-deployable PCR system was developed successfully. Our results showed that the assay was specific, sensitive, and reproducible, which would serve as an alternative method for preliminary screening and provide a valuable platform for the on-site detection of *Salmonella* and *Shigella*, especially in resource-limited and developing countries.

## Figures and Tables

**Table 1:** Primers and probes used in the iiPCR assay.

Primer/probe	Sequence (5′-3′)
Sal-F	GCT TGT ACT TTC CTT ATT C
Sal-R	AGY GYC AGC GAC CTG AAC
Sal-P	FAM-ACG CTA TTA GCT GTA AAA GAG CGC TG-BHQ1
Shi-F	TGA AGG AAA TGC GTT TCT
Shi-R	ACT TCT GAC CAT GGC TTC
Shi-P	HEX-TGT CGG GAG TGA CAG CAA ATG ACC TCC GCA-BHQ1

**Table 2:** Clinical evaluation of the iiPCR assay.

iiPCR	Bacterial culture		
	Positive	Negative	Total
Positive	64	4	68
Negative	0	115	115
Total	64	119	183

## Data Availability

The datasets generated during and/or analyzed during the current study are available from the corresponding author on reasonable request.
